# The Impact of Selective-Logging and Forest Clearance for Oil Palm on Fungal Communities in Borneo

**DOI:** 10.1371/journal.pone.0111525

**Published:** 2014-11-18

**Authors:** Dorsaf Kerfahi, Binu M. Tripathi, Junghoon Lee, David P. Edwards, Jonathan M. Adams

**Affiliations:** 1 Department of Biological Sciences, Seoul National University, Seoul, Republic of Korea; 2 School of Chemical and Biological Engineering, Interdisciplinary Program of Bioengineering, Seoul National University, Seoul, Republic of Korea; 3 Department of Animal and Plant Sciences, University of Sheffield, Sheffield, United Kingdom; University of Oxford, United Kingdom

## Abstract

Tropical forests are being rapidly altered by logging, and cleared for agriculture. Understanding the effects of these land use changes on soil fungi, which play vital roles in the soil ecosystem functioning and services, is a major conservation frontier. Using 454-pyrosequencing of the ITS1 region of extracted soil DNA, we compared communities of soil fungi between unlogged, once-logged, and twice-logged rainforest, and areas cleared for oil palm, in Sabah, Malaysia. Overall fungal community composition differed significantly between forest and oil palm plantation. The OTU richness and Chao 1 were higher in forest, compared to oil palm plantation. As a proportion of total reads, Basidiomycota were more abundant in forest soil, compared to oil palm plantation soil. The turnover of fungal OTUs across space, true β-diversity, was also higher in forest than oil palm plantation. Ectomycorrhizal (EcM) fungal abundance was significantly different between land uses, with highest relative abundance (out of total fungal reads) observed in unlogged forest soil, lower abundance in logged forest, and lowest in oil palm. In their entirety, these results indicate a pervasive effect of conversion to oil palm on fungal community structure. Such wholesale changes in fungal communities might impact the long-term sustainability of oil palm agriculture. Logging also has more subtle long term effects, on relative abundance of EcM fungi, which might affect tree recruitment and nutrient cycling. However, in general the logged forest retains most of the diversity and community composition of unlogged forest.

## Introduction

Tropical forests are one of the world’s most important reservoirs for biodiversity [1]. They contain an exceptional concentration of the world’s species, but are being reduced in area faster than any other ecosystem [2]. Roughly half of the world’s natural extent of tropical forest has been logged or converted to different land uses [3]. Some 403 million hectares of tropical rainforests have been included in timber estates and slated for selective logging [4], and between 2000 and 2010, approximately 13 million hectares of forest within the tropics were cleared for agricultural activities [5], [6], including oil palm plantations [7].

Anthropogenic disturbances in tropical forests are causing a dramatic decline in global biodiversity, and in associated biological processes that maintain the productivity and sustainability of ecosystems [8]. Several studies have shown that selective logging does not drastically impact the overall species richness and diversity of tropical forest [9]–[12], however, it has been shown that the impact of selective logging could be anything between fairly mild and severe depending on the intensity of logging [13] with changes in the composition of species, as forest-interior specialists decline and edge-tolerant, gap specialists increase in abundance [14], [15]. In contrast, the conversion of both primary and logged forest to agricultural land uses has been to shown to have a far greater negative impact on biodiversity than does logging. Conversion to agriculture results in a major reduction in biodiversity, again across a host of animal and plant taxa [16]. The conversion of primary and logged forest to agricultural plantations also results in a substantial decrease in the functional diversity of tropical ecosystems, with implications for the provision of ecosystem functions, whereas logging has lesser impacts on these metrics [17], [18]. As well as affecting plants and larger animals, land use change also affects the soil biota. Land use change affects soil pH, carbon and nutrient content [19], [20], causing shifts in soil microbial communities [21]–[23].

Fungi constitute one of the most diverse and dominant groups of organisms in soil, and they play important ecological roles in the ecosystem as decomposers, pathogens and plant mutualists [24], [25]. Understanding the structure and diversity of soil fungal communities is fundamental to the understanding of their function in the ecosystem and their impact on plant communities [26]. However, while minimal work has been done in the tropics to assess the effect of land use changes on soil bacterial communities [23], [27], until recently relatively little was known about the impacts of tropical land use change on soil fungal communities. Various studies have suggested that forest clearance to tree plantations or agricultural crops shifts soil fungal communities, linked to strong changes in soil properties [22], [28]. However, previous studies of forest clearance to other forms of agriculture on forest fungal communities have mostly been limited to techniques that give relatively low taxonomic resolution (i.e. T-RFLP and PCR-DGGE). Nevertheless, a recent study by McGuire et al. [29], which used high throughput sequencing to analyze soil fungal communities in Southeast Asian tropical forests in west Malaysia, showed that conversion of primary forest to oil palm plantations alters fungal community composition and function, whereas primary and logged forests were more similar in composition and nutrient cycling potential. However, there is a need for further studies to understand the impacts of logging cycles on soil fungal communities and of the conversion of logged forest to agriculture, since logged forests now dominate the tropics [3] and are much more likely to be converted to agriculture than primary forests [2], [6].

In this study, we also focused on the rainforests of the Sundaland region, of Southeast Asia, but some 1,800 km away in east Malaysia (Borneo). Across the Sundaland region, the primary forest has been subject to differing degrees of logging intensity. Much of the region’s forest (about 50%) has never been logged, while many areas have been subject to one or two logging cycles [30]. Also, oil palm is one of the most rapidly expanding crops in this region. This provides an opportunity to study the effect of different intensities of logging on the soil fungal community and also to evaluate if conversion of forest to oil palm plantation has a stronger impact on soil fungi than logging, as is the case for numerous macroscopic taxa. Our objective here was to understand whether land use change has an impact on the structure and diversity of fungal communities in the Yayasan Sabah (YS) logging concession in Malaysian, Borneo. We compared the fungal communities in forests with different logging histories (unlogged, once-logged and twice-logged), and oil palm plantations. We examined whether there are differences in α and â-diversity, as well as community composition. These results may provide important information for soil management policies, and estimation of ecological impact of land use change in this region.

## Materials and Methods

### Study area

The study area is located within the Yayasan Sabah (YS) logging concession and contiguous oil palm plantation areas, in Sabah, Malaysian Borneo (4°58′ N, 117°48′ E). The forests in this area are naturally dominated by valuable timber tree species belonging to the family Dipterocarpaceae [31]. Due to logging for the wood industry and clearance for palm oil plantations, the area of forest in Borneo - as elsewhere in the tropics - has been dramatically reduced in recent decades [32].

Fieldwork was conducted in the Ulu Segama-Malua Forest Reserve (US-MFR) and adjacent oil palm estates in Sabah, Borneo. Some areas of forest were logged between 1970 and 1990, and some of these were then re-logged between 2000 and 2007. During the first logging rotation, approximately 113 m^3^ per hectare (range 73 m^3^ to 166 m^3^) of commercially valuable trees >0.6 m diameter were extracted. During the second logging rotation, an additional 31 m^3^ per hectare (range 15 m^3^ to 72 m^3^) of timber were removed [10], [31]. Selectively logged forest in the US-MFR is adjacent to the 45,200 ha Danum Valley Conservation Area (DVCA) and Palum Tambun Watershed Reserve, containing large areas of unlogged forest [10], [16]. Oil palm plantations are situated to the north and south of the US-MFR, with mature palms of 20 to 30 years old, planted at a density of 100 trees per hectare.

### Soil sampling and DNA extraction

From September to October 2012, twenty-four transects each of 200 m in length were located across four different land uses: unlogged (primary) forests, once-logged and twice-logged forests, and oil palm plantations, with six transects per habitat. Within each habitat, distances between transects ranged from 500 m to 65 km, whereas across habitats, distances between transects ranged from 1 km to 67 km. From each transect, at 50 m intervals, approximately 50 g from the top 5 cm of soil (excluding the leaf litter layer) was taken in a sterile plastic bag using a trowel, giving five samples of soil per transect. The trowels were thoroughly cleaned with ethanol between successive transect sampling. All soil samples were then sieved (2 mm) in laboratory to homogenize the sample and stored at –20°C until DNA extraction [10]. Twenty-four soil DNA extractions (one for each transect) were performed using 0.3 g of soil, with the Power Soil DNA extraction kit (MO BIO Laboratories, Carlsbad, CA, USA) following the directions described by the manufacturer.

### PCR amplification and pyrosequencing

Fungal DNA was amplified using ITS primers targeting the internal transcribed spacer (ITS) region 1 and 2. Forward primers comprised the 454 Fusion Primer A-adaptor, a specific multiplex identifier (MID) barcode, and the ITS1F primer (5′-CTTGGTCATTTAGAGGAAGTAA-3′) [33], while the reverse primer was composed of the B-adapter and ITS4 primer (5′-TCCTCCGCTTATTGATATGC-3′) [34]. Polymerase chain reactions (PCR) were performed in 50 µl reactions using the following temperature program: 95°C for 10 min s; 30 cycles of 95°C for 30 s, 55°C for 30 s, 72°C for 30 s; and 72°C for 7 min. The PCR products were purified using the QIAquick PCR purification kit (Qiagen) and quantified using PicoGreen (Invitrogen) spectrofluorometrically (TBS 380, Turner Biosystems, Inc. Sunnyvale, CA, USA). 50 ng of purified PCR product for each sample were combined in a single tube and sent to Macrogen Inc. (Seoul, Korea) for sequencing using 454/Roche GS FLX Titanium Instrument (Roche, NJ, USA).

### Sequence processing

Initial quality filtering and denoising were performed following the 454 SOP in the mothur pipeline [35]. The ITS1 region was verified and extracted using the ITS1 extractor for fungal ITS sequences [36]. Putative chimeric sequences were detected and removed via the Chimera Uchime algorithm contained within mother [37]. Operational taxonomic units (OTUs) were assigned using the QIIME implementation of UCLUST [38], with a threshold of 97% pairwise identity. OTUs were classified taxonomically using the classify command in mothur at 80% Naïve Bayesian bootstrap cutoff with 1000 iterations against the UNITE database [39]. Ectomycorrhizal (EcM) fungi were determined by matching taxonomy assignments with established EcM lineages as determined by recent phylogenetic and stable isotope data [40]. The 454 sequence run has been deposited in the NCBI Sequence Read Archive under accession number SRP041467.

### Statistical analysis

To correct for differences in number of reads, which can bias diversity estimates, all samples were rarified to 3,347 reads per sample. To test for effects of land use types on the OTU richness and diversity indices, we used a linear model (LM) for normal data or generalized linear model (GLM) for non-normal data, considering land use as the major factor. We used the same procedure to test whether relative abundance of the most abundant phyla differed among different land use types. We also assessed the effect of land use on the relative abundance at the order and genus levels within those phyla that showed significant differences due to land use. Post-hoc Tukey tests were used for pairwise comparisons. When neither a linear nor a generalized linear model fitted the data, we used a Kruskall-Wallis test to assess the effect of land use on the relative abundance of fungal taxa, with the Bonferroni correction to assess pairwise comparisons.

To test whether species composition results may have been influenced by pseudoreplication within study sites, we used a Mantel test (Mantel Nonparametric Test Calculator 2.0) [41] to compare transect matrices of fungal compositional to geographic distance between pairs of transects within a site and between pairs of transects across the entire dataset [42], [43].

The OTU-based community similarity was calculated by using the Bray-Curtis index [44]. Non-metric multidimensional scaling (NMDS), to visualize the change in species composition across the land use types, was conducted in Primer-E software (Version 6, Plymouth, UK). Then, we tested the difference among different land use types using an analysis of similarity (ANOSIM).

We measured β-diversity amongst land use types following Anderson et al. [45], which is defined as the variation in community structure without defining a particular gradient or direction. Therefore, we estimated true β-diversity following Whittaker [46] in [47] for every land use type. In addition, we calculated β-diversity as the average distance from each site to the group centroid [45]. The betadisper function in R was used to test if β-diversity shows any difference between land use types. True β-diversity (i.e. 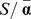) for each pair of samples within each of the four land uses was estimated by the following equation:
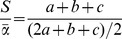



Where *S* is the total number of OTUs in two samples, 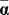 is the average number of OTUs for both samples, *a* is shared OTUs between both samples, *b* is OTUs found only in sample 1 and *c* are OTUs found only in sample 2. To compare true β-diversity among land uses, we used a linear model using land use as factor, and sample as random factor to control for pseudoreplication, as every sample is used in more than one comparison within each land use. Post-hoc Tukey tests were used for pairwise comparisons among different land uses.

## Results

**Figure 1:**
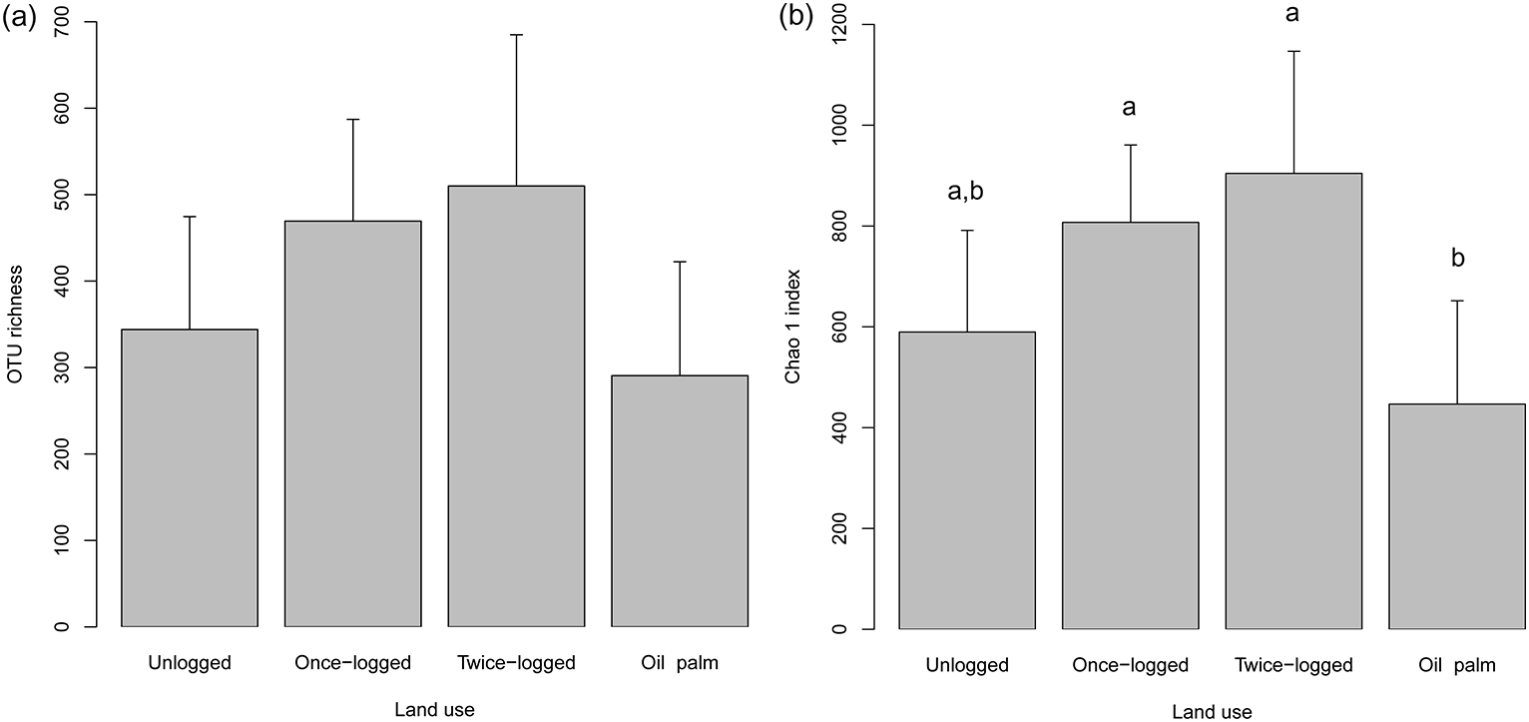
Diversity indices of the fungal community across different land uses in Sabah, Malaysian Borneo. (a) OTU richness and (b) Chao1 index. Pairwise comparisons are shown; different letters denote significant differences between groups at P<0.05.

A total of 114,744 quality sequences were obtained from the 24 soil samples, with coverage ranging from 3,347–6,456 sequences. After rarifying to 3,347 reads per sample, we obtained a total of 80,328 sequences, and of these around 84% sequences were classified up to phylum level with a total of 5,327 OTUs (defined at ≥97% sequence similarity level). Fungal OUT

richness (i.e. number of OTUs) was marginally significantly different across land use types (F_3,24_ = 2.98, P = 0.05; Fig. 1a), with lowest levels of OTU richness observed in oil palm plantations compared to primary and logged forests. Predicted OTU richness calculated using the Chao1 estimator was significantly higher in logged forests than oil palm plantations (F3,24 = 4.74, P = 0.01; Fig. 1b), whereas Shannon index did not show any variation among land uses (F3,24 = 1.93, P = 0.15).

**Figure 2:**
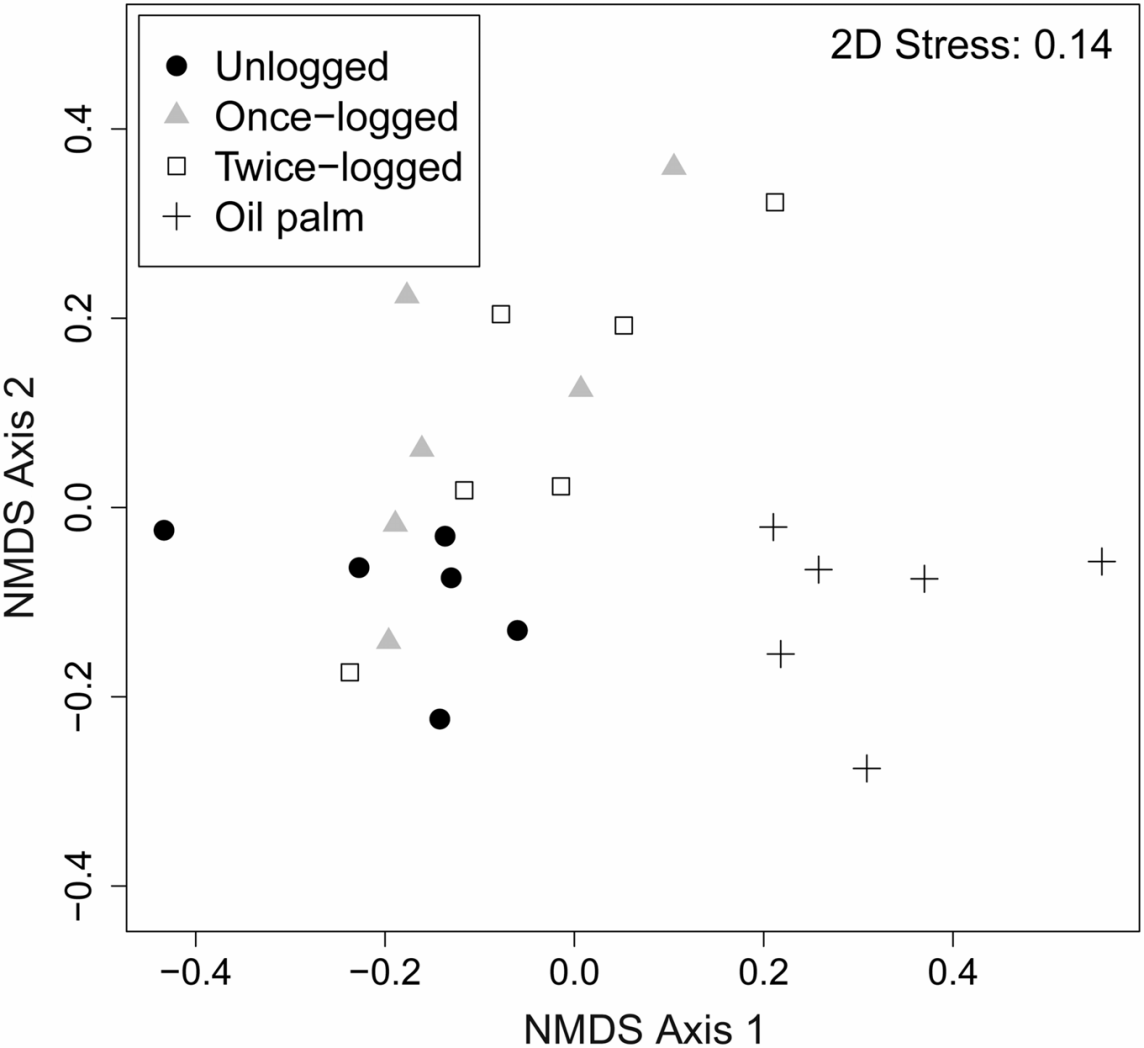
Non-metric multidimensional scaling (NMDS) ordination showing clustering of fungal communities among different land uses in Sabah, Malaysian Borneo.

**Figure 3:**
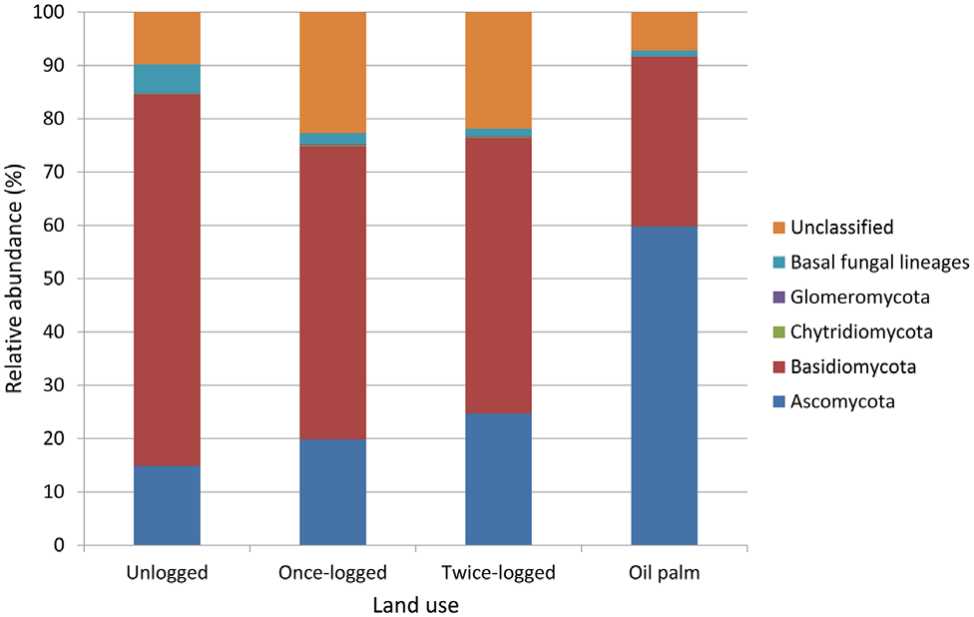
Relative abundance of dominant fungal phyla among different land uses in Sabah, Malaysian Borneo.

The NMDS plots of pairwise Bray–Curtis dissimilarities showed that fungal communities were clustered significantly across land use types (ANOSIM: R = 0.51, P<0.001; Fig. 2). Mantel tests showed no effect of distance on the composition of fungal communities across different land use types in Borneo (all P>0.07). The majority of fungal sequences recovered in our study belonged to the Basidiomycota and Ascomycota, with relative abundances of 52% and 29%, respectively (Fig. 3). The basal fungal lineages represented 2%, followed by Glomeromycota and Chytridiomycota with less than 1%, and 15% of the detected sequences were unclassified (Fig. 3). We found a significant change in the relative abundance of the two most dominant phyla: Basidiomycota (F_3,24_ = 4.23, P = 0.01) and Ascomycota (F_3,24_ = 4.81, P = 0.01) along different land uses. The abundance of Basidiomycota was greater in the unlogged forest, intermediate in the logged forest, and least in the oil palm. Conversely, the oil palm plantations had greater abundance of Ascomycota compared to forest soils (Fig. 3). At the order level, there were significant differences in the relative abundances of the most dominant orders (Table 1). The results revealed that the relative abundance of *Agaricales*, *Russulales*, *Thelephorales*, *Trichosporonales, Sebacinales* and *Helotiales* were significantly higher in the forest than oil palm plantations (P<0.05; Table 1). However, *Hypocreales*, *Sporidiobolales* and *Pleosporales*, were more abundant in oil palm plantations than unlogged and logged forests (P<0.05; Table 1).

**Table 1:** Comparison of relative abundance of the dominant fungal orders within the phyla Ascomycota and Basidiomycota among land uses^a^.

Taxa	F or χ^2b^	df	P	Pairwise comparisons^c^
**Ascomycota**				
*Helotiales*	3.67	3, 24	0.02	Once-logged > oil palm
*Hypocreales*	5.54	3, 24	0.006	Once-logged/twice-logged/unlogged < oil palm
*Pleosporales*	6.42	3, 24	0.003	Once-logged/unlogged < oil palm
**Basidiomycota**				
*Agaricales*	10.9	3, 24	0.0001	Once-logged/twice-logged/unlogged > oil palm
*Russulales*	11.3	3, 24	0.0001	Unlogged > Once-logged/twice-logged/oil palm
*Sebacinales*	11.1*	3	0.01	Unlogged/twice-logged > oil palm
*Sporidiobolales*	15.1*	3	0.001	Once-logged/twice-logged/unlogged < oil palm
*Thelephorales*	10.0*	3	0.01	Unlogged/twice-logged > oil palm
*Trichosporonales*	13.3	3, 24	0.0001	Once-logged/twice-logged/unlogged > oil palm

a Only orders for which significant differences were found are shown.

b Effect of land use on relative abundance evaluated by linear or generalized linear model or by the Kruskal-Wallis test (*).

c Pairwise comparisons by *post hoc* Tukey test for linear/generalized linear models or *P* values Bonferroni-corrected for Kruskal-Wallis. Differences were considered significant at a P value of <0.05.

**Figure 4:**
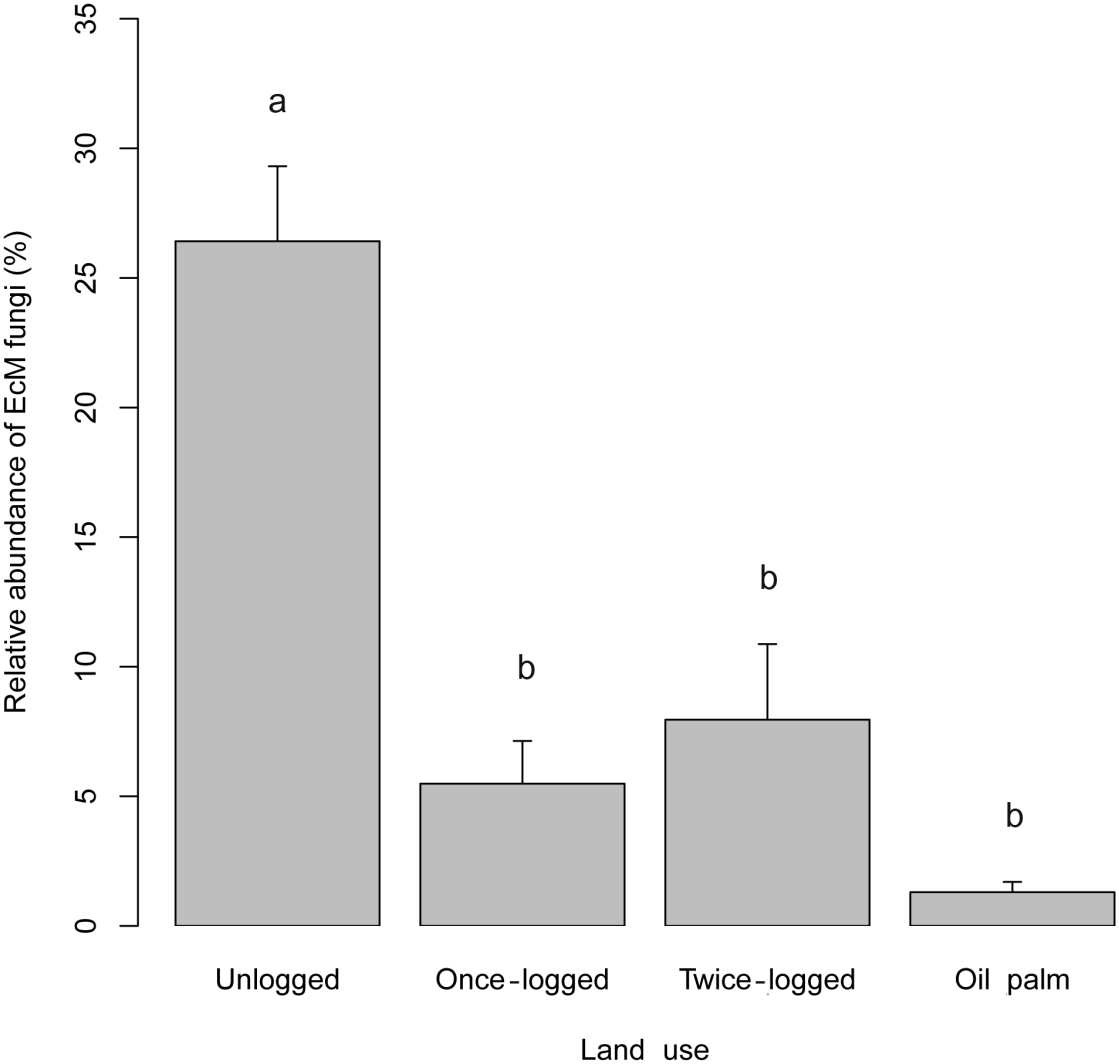
Relative abundance (means ± SD) of ectomycorrhizal (EcM) fungal sequences among different land uses in Sabah, Malaysian Borneo. Pairwise comparisons are shown; different letters denote significant differences between groups at P<0.05.

A total of 11,421 sequences belonged to known groups of ectomycorrhizal (EcM) fungi, with the EcM fungi representing around 10% (11,421 sequences) of the total detected fungal sequences, with 180 OTUs. The relative abundance of EcM sequences was significantly different across land use types, with highest and lowest relative abundances observed in primary forest (mean relative abundance = 26%) and oil palm plantations (mean relative abundance = 0.5%), respectively (χ^2^ = 18.04, P<0.001; Fig. 4). From our soil samples, we identified 14 genera belonging to EcM fungi with *Russula* as the most dominant genus (65% of total EcM sequences), followed by *Tomentella*, *Sebacina* and *Lactarius*. The relative abundance of these four dominant EcM genera combined were significantly higher in forest soils compared to oil palm plantations (P<0.05). For *Russula* alone, we also found a difference in abundance between unlogged (greatest abundance), once-logged (less abundant), and twice-logged forest (lowest abundance of *Russula*) (P<0.05 in each case).

**Figure 5:**
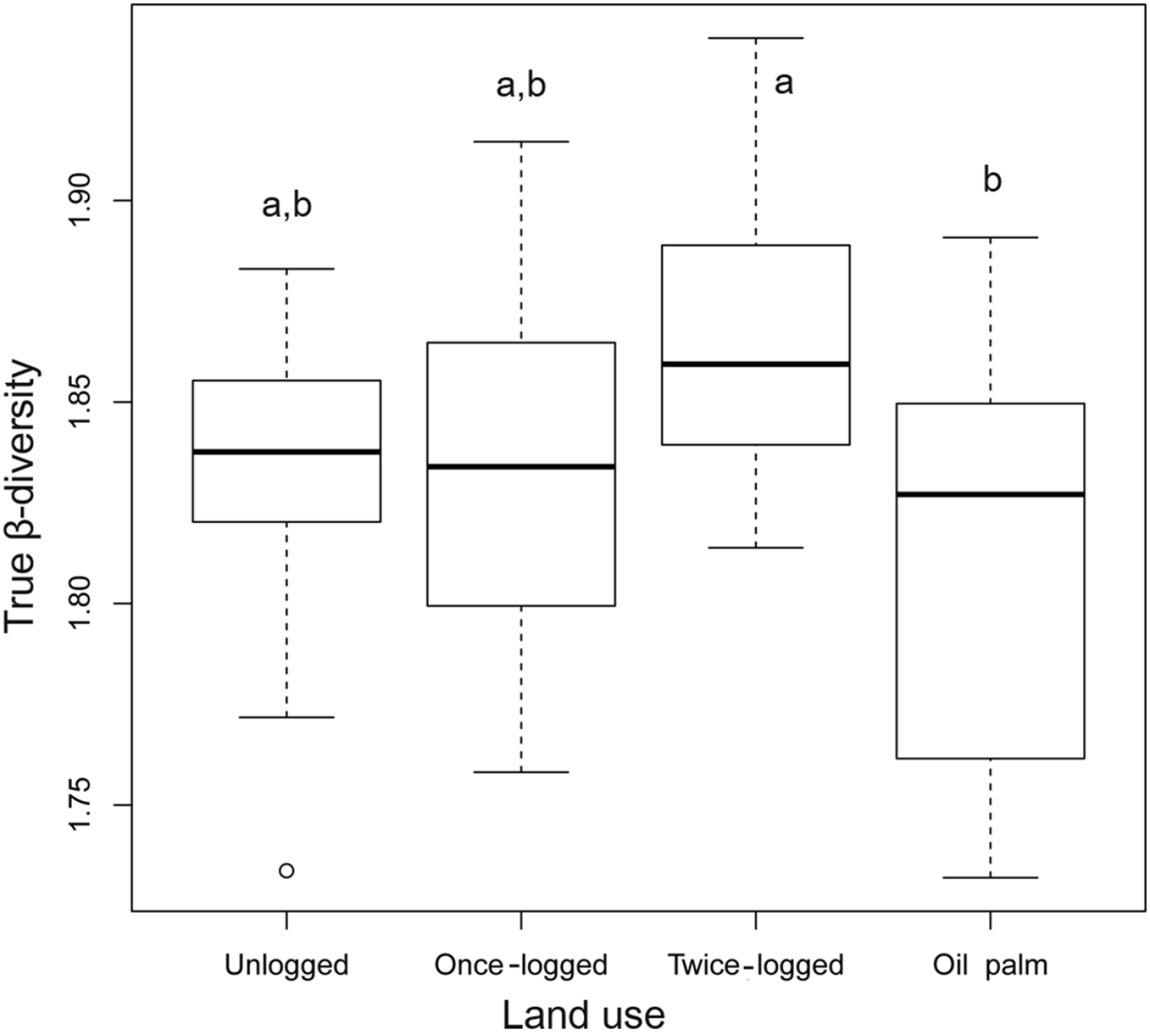
Fungal community true β-diversity (i.e. 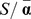
 **) among the four land uses in Sabah, Malaysian Borneo.** Boxes show the lower quartile, the median and the upper quartile. Pairwise comparisons are shown; different letters denote significant differences between groups at P<0.05.

The β-diversity, measured as the average distance of all samples to the centroid in each land use type, did not show significant difference among land uses (F_3,24_ = 2.77, P = 0.06). However, there was a significant effect of forest conversion to oil palm plantations on fungal true β-diversity (i.e. 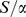; F_3,24_ = 3.85, P = 0.01), with oil palm having lowest true β-diversity compared to unlogged and logged forests (Fig. 5). Logging did not produce any significant change in true β-diversity.

## Discussion

Our results showed that fungal OTU richness and Chao1 index differed among land uses. Oil palm plantations had lower OTU richness, which can be attributed to the effects of anthropogenic intervention and forest conversion. This contrasts with the findings on bacterial communities of Lee-Cruz et al. [27] in the same study site, where they found that OTU richness and diversity indices did not differ among land uses, and that α-diversity was similar in forests and oil palm plantations.

We found that the structure of fungal communities differed most fundamentally between forests and oil palm plantations. This finding mirrors that of McGuire et al. [29], who found that the community of soil fungi collected across three different land uses in Malaysia differed between oil palm plantations and forests. Changes in fungal community composition in logged forest and former forest areas could directly impact the functioning of soil communities and their ability to provide key ecosystem services, such as decomposition and nutrient recycling [22]. Given that fertilizer prices are predicted to rise dramatically in the coming decades [48], these results suggest that improvements in agricultural methods by establishing diversified farms could be necessary for sustaining vital soil biodiversity and ecosystem-service values.

The abundance pattern of fungal taxa detected here is similar to the soils of the Neotropics and elsewhere, where Basidiomycota and Ascomycota are also the most prevalent groups [49]–[53]. The most abundant orders of Basidomycota in the forest areas we sampled were *Russulales*, *Agaricales*, *Sporidiobolales*, *Telephorales*, *Trichosporonales* and *Sebacinales*. They all became less abundant in oil palm plantations compared to primary forest. These orders are generally reported to be the most varied and abundant groups of fungi in forests around the world [40], [54], [55], they have also been characterized as being lignicolous, saprobic or mycorrhizal, associated with litter decomposition, and they are known to degrade plant-derived cellulose [56]. In our forest plots, the ectomycorrhizal orders *Russulales* and *Telephorales* were the most common. This pattern has also been found in forests from the Neotropics and African tropics [57]–[59].

The lower abundance of Basidiomycota in oil palm soils may be due to the lack of large quantities of coarse woody debris, often derived from roots or branches of forest trees, in oil palm plantations. However, it may be worth investigating whether the change in abundance of Basidiomycota - and change in their overall community structure - with conversion of forest to oil palm may have implications for long term nutrient processing in the oil palm soils. Since Basidiomycota as a group are often able to break down relatively recalcitrant substrates and changes in their abundance and community composition might impede nutrient recycling in oil palm soils. The converse increase in Ascomycota, may be seen in terms of the relatively lignin-poor and nutrient-rich character of most of the organic matter reaching the soil from roots and leaves of oil palm, and from the herbaceous weedy layer that grows under the palms.

We found that both a history of logging, and forest conversion to oil palm plantation, resulted in shifts in EcM fungal communities. The most drastic effects were with forest conversion to oil palm. However, logging history also had detectable effects EcM fungi relative abundance. Ectomycorrhizas are one of the most important widespread types of mycorrhiza in forests of the cool temperate and boreal latitudes [60], and they also form an important group and often the dominant ecologically and economically important minority of Dipterocarpaceae family trees in tropical Asia [61]–[65].

The lower abundance of EcM fungi in logged forests might be due to a thinner, more incomplete root mat following past logging disturbance. The much lower abundance of EcM fungi in oil palm plantation soils could be partly due to the much lower abundance of potential host roots (e.g. Dipterocarpaceae which are absent from the palm plantations). Such fungi have generally been found to recover slowly from disturbance even when potential host plants are present [66]. Among the detected genera in our study, we found that *Russula*, *Sebacina*, *Lactarius* and *Tomentella* showed significant impact of land use change. *Russula* was relatively the most abundant EcM genus in our samples: this genus has been found to be common on roots of dipterocarp forests, tropical and southern hemisphere angiosperm forests [62], [67], [68].

This study also investigated the impacts of land use change on the turnover of fungal communities across space (β-diversity), and results suggest that there is a spatial homogenization of fungal communities in oil palm agriculture compared to forest: fungal β-diversity in oil palm plantations was lower than forest. Habitat conversion to agriculture also reduces β-diversity of soil bacteria in Amazonia [69]. In contrast, across the same land use system and sites in Borneo as we studied here, there was actually an increase in β-diversity of bacteria with both logging and conversion to oil palm [27].

There are some caveats that accompany our findings. We worked in only one biogeographic region and on only one form of agriculture. It is thus important to replicate this work in other logging systems and in other key expanding crops, including soya, sugar cane, and cacao. We also did not explicitly demonstrate the impacts of changing fungal composition on soil ecosystem functions and services: this is a major knowledge gap, with critical importance to the development of sustainable logging and, in particular, agricultural systems in the tropics.

In conclusion, the conversion of both primary and logged forest to oil palm drives a change in the overall fungal community, including EcM fungi abundance, and an associated decrease in total fungal community beta-diversity. This finding invites further studies that investigate the long-term implications of such changes for agricultural sustainability. There was a more subtle long-term impact of logging on fungal communities. Most measurable features of the unlogged forest fungal community remained unchanged after logging. However, there were significant changes in EcM fungal abundance due to logging, which could have a pervasive impact since EcM fungi are thought to play a key role in tree growth and community structure. Despite this, the lack of drastic changes in the overall forest fungal community structure following logging strengthens the view that logged forest is not necessarily an irretrievably damaged and drastically altered system, and that protecting it from conversion to oil palm may still have considerable conservation benefits.
